# Supplementary material for: Does a waiting room increase same-day treatment for sexually transmitted infections among pregnant women? A quality improvement study at South African primary healthcare facilities
Source: BMC Health Serv Res. 2025 Apr 4;25:501. doi: 10.1186/s12913-025-12607-x (PMC11971735; doi:10.1186/s12913-025-12607-x)

**Additional file 1 – Socio-economic and health characteristics of included and excluded participants**


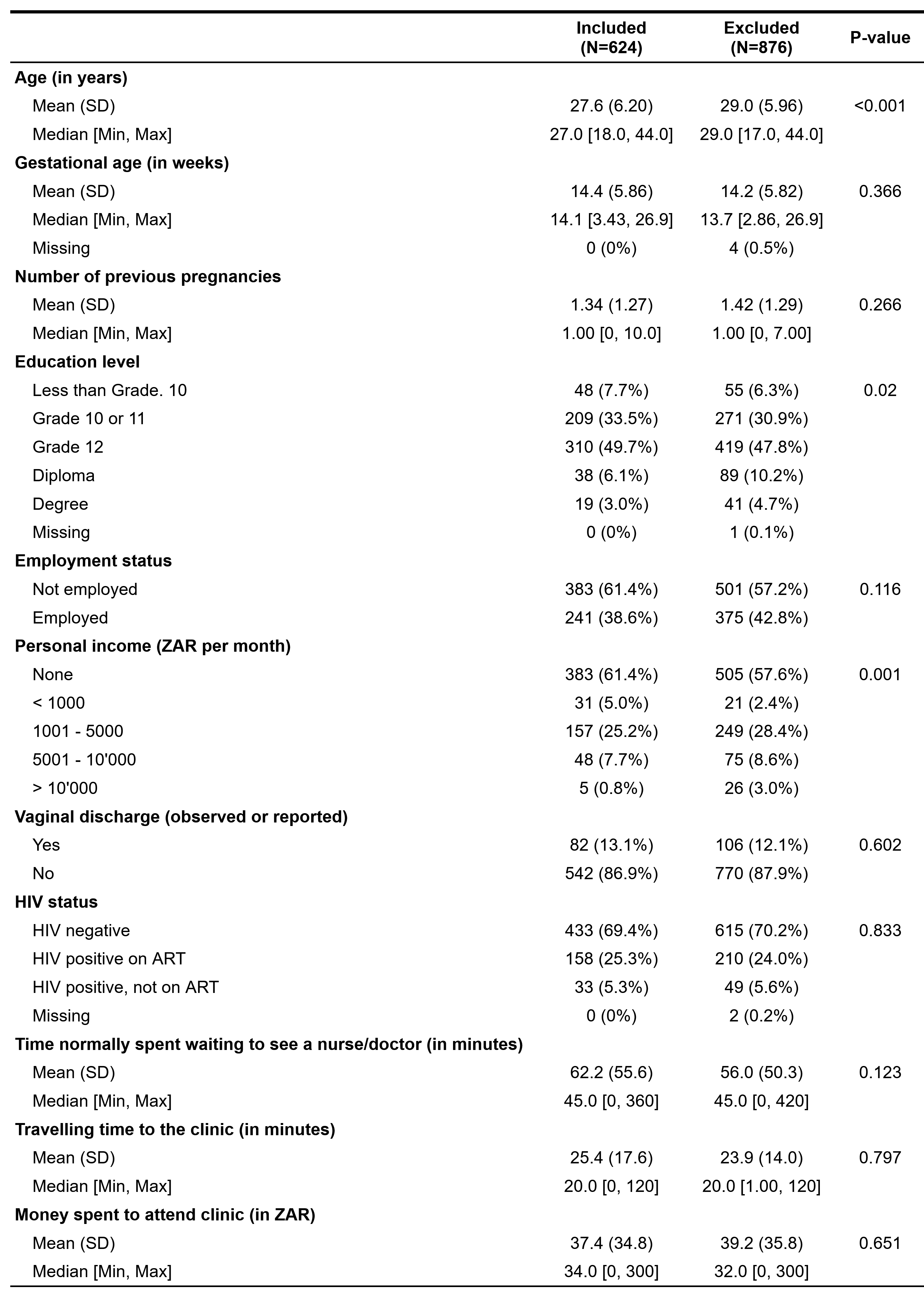

Supplement: Supplementary file 1 — Additional file 1. Socio-economic and health characteristics of included and excluded participants [file 12913_2025_12607_MOESM1_ESM.docx]
