# Supplementary material for: Does a waiting room increase same-day treatment for sexually transmitted infections among pregnant women? A quality improvement study at South African primary healthcare facilities
Source: BMC Health Serv Res. 2025 Apr 4;25:501. doi: 10.1186/s12913-025-12607-x (PMC11971735; doi:10.1186/s12913-025-12607-x)
Supplement: Supplementary file 2 — Additional file 2. Percentage of women who waited for results by clinic and intervention date (14 December 2023) [file 12913_2025_12607_MOESM2_ESM.docx]

**Additional file 2 – Percentage of women who waited for results by clinic and intervention date (14 December 2023)**

| Waited for results,  % (95% CI)  n/N | Clinic A | Clinic B | Clinic C | Clinic D | Clinic E | Overall |
| --- | --- | --- | --- | --- | --- | --- |
|  | Intervention:  waiting room  introduced | | No intervention:  always had a  waiting room | | No intervention: never had a waiting room |  |
| Pre-intervention | 26 (16-39) 17/65 | 10 (4-18) 8/84 | 94 (86-98) 77/82 | 68 (43-86) 13/19 | 18 (12-26) 21/118 | 37 (32-42) 136/368 |
| Post-intervention | 18 (9-32) 9/50 | 2 (0-12) 1/49 | 80 (66-90) 41/51 | 69 (49-84) 20/29 | 23 (15-35) 18/77 | 35 (29-41) 89/256 |
| Total | 23 (16-32) 26/115 | 7 (3-13) 9/133 | 89 (82-93) 118/133 | 69 (54-81) 33/48 | 20 (15-26) 39/195 | 36 (32-40) 225/624 |
