# Supplementary material for: Does a waiting room increase same-day treatment for sexually transmitted infections among pregnant women? A quality improvement study at South African primary healthcare facilities
Source: BMC Health Serv Res. 2025 Apr 4;25:501. doi: 10.1186/s12913-025-12607-x (PMC11971735; doi:10.1186/s12913-025-12607-x)
Supplement: Supplementary file 3 — Additional file 3. Secondary analysis 1: All clinics, grouped by waiting room availability [file 12913_2025_12607_MOESM3_ESM.docx]

**Additional file 3 – Secondary analysis 1: All clinics, grouped by waiting room availability**

|  | | Waiting room vs. no waiting room |
| --- | --- | --- |
| Absolute percentage difference,  % (95% confidence interval) | | +39 (+31 to +47) |
| Adjusted absolute percentage difference,  % (95% confidence interval) | |  |
|  | Adjusted for employment status of participants  + STI positivity  + proportions of symptomatic participants  + load shedding  (in 3 categories: no load shedding, stage 1-3, stage 4-6)  + the distance in metres to the nearest food shop  + work experience of the nurse  (in 3 categories: 9 years=low, 15 years=middle, 33 years=long) | +39 (+31 to +47)  +41 (+33 to +49)  +39 (+31 to +47)  +42 (+35 to +50)  +7 (–4 to +18)  –8 (–15 to –0.3) |
