# Supplementary material for: Does a waiting room increase same-day treatment for sexually transmitted infections among pregnant women? A quality improvement study at South African primary healthcare facilities
Source: BMC Health Serv Res. 2025 Apr 4;25:501. doi: 10.1186/s12913-025-12607-x (PMC11971735; doi:10.1186/s12913-025-12607-x)
Supplement: Supplementary file 4 — Additional file 4. Sub-group analysis: Clinics A and B combined, grouped by presence of a sexually transmitted infection [file 12913_2025_12607_MOESM4_ESM.docx]

**Additional file 4 – Sub-group analysis: Clinics A and B combined, grouped by presence of an STI**

|  | | Percentage waited, % (95% CI, n/N) | Absolute percentage difference, % (95% CI) | Adjusted^1^ absolute percentage difference, % (95% CI) | |
| --- | --- | --- | --- | --- | --- |
| No STI | | 13 (7-21, 13/101) |  |  |  |
|  | Pre-intervention | 18 (7-35, 6/34) |  |  |  |
|  | Post-intervention | 10, (4-20, 7/70) | –9 (–26 to –7) | –11 (–29 to +7) | |
| STI present | | 15 (10-22, 22/147) |  |  |  |
|  | Pre-intervention | 16 (10-24, 19/118) |  |  |  |
|  | Post-intervention | 10 (3-29, 3/29) | –6 (–20 to –8) | –5 (–23 to +12) | |
| p-value for interaction between STI presence and absence | |  | 0.79 | 0.76 | |
| Legend: CI, 95% confidence interval; 1, adjusted for employment status of participants, STI positivity, proportions of symptomatic participants, load shedding (in three categories: no load shedding, stage 1-3, stage 4-6), and the distance in metres to the nearest food shop | | | | | |
