# Supplementary material for: Does a waiting room increase same-day treatment for sexually transmitted infections among pregnant women? A quality improvement study at South African primary healthcare facilities
Source: BMC Health Serv Res. 2025 Apr 4;25:501. doi: 10.1186/s12913-025-12607-x (PMC11971735; doi:10.1186/s12913-025-12607-x)
Supplement: Supplementary file 5 — Additional file 5. [file 12913_2025_12607_MOESM5_ESM.docx]

**Additional file 5 – Reasons for not waiting for STI test results, or for changing intention, by clinic**

|  | **Clinic A** | **Clinic B** | **Clinic C** | **Clinic D** | **Clinic E** | **Overall** |
| --- | --- | --- | --- | --- | --- | --- |
| Total number of participants | 115 | 133 | 133 | 48 | 195 | 624 |
| Responded to questions about waiting for results, n (%) | 74 (64) | 77 (58) | 76 (58) | 40 (83) | 125 (64) | 392 (63) |
| **“Are you planning to wait for your results today?”** |  |  |  |  |  |  |
| Yes | 48 (65) | 7 (9) | 65 (86) | 35 (88) | 20 (16) | 175 (45) |
| No | 26 (35) | 70 (91) | 11 (15) | 5 (13) | 105 (84) | 217 (55) |
|  |  |  |  |  |  |  |
| **“What is your main reason why you are not intending to wait today?”** | **Clinic A (n=26)**  **n (%)** | **Clinic B (n=70)  n (%)** | **Clinic C (n=11)**  **n (%)** | **Clinic D (n=5)**  **n (%)** | **Clinic E (n=105) n (%)** | **Overall (n=217) n (%)** |
| Have to get back to my kids/family | 5 (19) | 13 (19) | 3 (27) | 2 (40) | 43 (41) | 66 (30) |
| Have to get to work/school | 8 (31) | 18 (26) | 5 (46) | 1 (20) | 28 (27) | 60 (28) |
| Hungry | 5 (19) | 12 (17) | 0 | 1 (20) | 11 (11) | 29 (13) |
| Want to go to the shop | 0 | 9 (13) | 1 (9) | 0 | 16 (15) | 26 (12) |
| Not feeling well | 2 (8) | 8 (11) | 0 | 0 | 1 (1) | 11 (5) |
| No time/going somewhere | 0 | 5 (7) | 1 (9) | 0 | 2 (2) | 8 (4) |
| Load shedding | 3 (12) | 2 (3) | 0 | 0 | 0 | 5 (2) |
| Referred to hospital | 1 (4) | 1 (1) | 0 | 0 | 2 (2) | 4 (2) |
| Too hot | 0 | 2 (3) | 0 | 0 | 0 | 2 (1) |
| Boring | 0 | 0 | 0 | 0 | 2 (2) | 2 (1) |
| Transport availability | 0 | 0 | 1 (9) | 0 | 0 | 1 (1) |
| No space to wait | 1 (4) | 0 | 0 | 0 | 0 | 1 (1) |
| Didn't come alone to the clinic | 0 | 0 | 0 | 1 (20) | 0 | 1 (1) |
| Tired | 1 (4) | 0 | 0 | 0 | 0 | 1 (1) |
|  |  |  |  |  |  |  |
| **“What would make you change your mind?”** | **Clinic A (n=26)**  **n (%)** | **Clinic B (n=70)  n (%)** | **Clinic C (n=11)**  **n (%)** | **Clinic D (n=5)**  **n (%)** | **Clinic E (n=105) n (%)** | **Overall (n=217) n (%)** |
| Nothing | 20 (77) | 28 (40) | 11 (100%) | 3 (60) | 95 (91) | 157 (72) |
| If I did not have other commitments/not be in a hurry | 2 (8) | 23 (33) | 0 | 0 | 1 (1) | 26 (12) |
| Food | 3 (12) | 10 (14) | 0 | 1 (20) | 9 (9) | 23 (11) |
| If I had someone looking after the kids | 0 | 5 (7) | 0 | 1 (20) | 0 | 6 (3) |
| If I can feel better | 0 | 3 (4) | 0 | 0 | 0 | 3 (1) |
| Comfortable waiting space | 1 (4) | 1 (1) | 0 | 0 | 0 | 2 (1) |
|  |  |  |  |  |  |  |
| **“What made you change your mind about waiting for the results?”** | **Clinic A (n=1), n (%)** | **Clinic B (n=0), n (%)** | **Clinic C (n=1), n (%)** | **Clinic D (n=2), n (%)** | **Clinic E (n=7, n (%)** | **Overall (n=11) n (%)** |
| Waiting for other clinic procedures (for example medication) | 1 (100) | 0 | 0 | 0 | 6 (86) | 7 (64) |
| Education about importance of waiting by the nurse | 0 | 0 | 1 (100) | 2 (100) | 0 | 3 (27) |
| Returned to clinic after being called | 0 | 0 | 0 | 0 | 1 (14) | 1 (9) |
